# Association of a novel nutritional index, the triglyceride-cholesterol-body weight index (TCBI), with incident mild cognitive impairment: CHARLS 2011–2018

**DOI:** 10.3389/fnut.2026.1834512

**Published:** 2026-06-04

**Authors:** Yiwen Lu, Dongyang Jiang, Jinfeng Zhang, Chunxiang Wang, Jihong Li, Ying Liang

**Affiliations:** 1 School of Clinical Medicine, Shandong Second Medical University, Weifang, China; 2 Department of General Practice, The First Affiliated Hospital of Shandong First Medical University & Shandong Provincial Qianfoshan Hospital, Jinan, China; 3 Graduate School of Shandong First Medical University, Shandong Academy of Medical Sciences, Jinan, China

**Keywords:** CHARLS, cohort studies, mild cognitive impairment, nutritional index, TCBI

## Abstract

**Background:**

The triglyceride-cholesterol-body weight index (TCBI) is a recently proposed nutritional index derived from lipid measures and body weight. Although malnutrition has been linked to cognitive decline in middle-aged and older adults, longitudinal population-based evidence on the association between TCBI and incident mild cognitive impairment (MCI) remains limited. This study examined the association between TCBI and incident MCI in middle-aged and older adults, while accounting for death as a competing event.

**Methods:**

Data were obtained from the China Health and Retirement Longitudinal Study (CHARLS, 2011–2018). After applying inclusion and exclusion criteria, the final analytical sample consisted of 5,510 participants. The TCBI was calculated as follows: TCBI = [TC (mg/dL) × TG (mg/dL) × body weight (kg)] / 1,000. Log10-transformed TCBI was analyzed as a continuous variable, per 1-SD increment, and by quartiles. Cox proportional hazards models were used to estimate the association between TCBI and incident MCI, with sequential adjustment for demographic, lifestyle, and clinical metabolic covariates. Restricted cubic splines were used to assess nonlinearity. Threshold effects were further examined using piecewise Cox models, and model fit was compared with likelihood ratio tests. Sensitivity analyses included the Fine–Gray subdistribution hazard model with all-cause mortality as a competing event, and Cox regression analyses excluding participants with baseline dyslipidemia.

**Results:**

Over the 7-year follow-up, 1,063 incident cases of MCI (19.29%) and 437 deaths (7.93%) were recorded. In fully adjusted multivariable models, each 1-unit increase in log10-transformed TCBI (Lg TCBI) was associated with a reduced risk of MCI (HR = 0.73, 95% CI: 0.57–0.94, *P* = 0.014); similarly, each 1-SD increase in Lg TCBI was associated with a lower risk (HR = 0.91, 95% CI: 0.84–0.98, *P* = 0.014). When Lg TCBI was categorized into quartiles, participants in Q3 (HR = 0.76, 95% CI: 0.63–0.92, *P* = 0.005) and Q4 (HR = 0.75, 95% CI: 0.61–0.93, *P* = 0.007) exhibited significantly lower risks of MCI compared to Q1. However, no significant difference was observed between Q2 and Q1 (*P* >0.05). These findings were consistent in analyses that accounted for all-cause mortality as a competing event and in analyses excluding participants with baseline dyslipidemia. Restricted cubic spline analysis suggested a non-linear association between TCBI and incident MCI. The piecewise Cox model fit the data better than the linear model (likelihood ratio test *P* = 0.013). No significant effect modification was observed in subgroup analyses.

**Conclusion:**

Lower TCBI levels were associated with a higher risk of incident MCI in middle-aged and older adults. As a simple and practical index derived from routine lipid measurements and body weight, TCBI may help identify individuals at increased risk of cognitive decline in primary care and community settings.

## 1 Introduction

As populations age worldwide, the burden of cognitive impairment continues to increase (1). Cognitive impairment encompasses a spectrum of conditions, including mild cognitive impairment (MCI) and dementia. MCI represents an intermediate stage between normal cognitive aging and dementia, characterized by an increased risk of progression to dementia (2–4). Pathological brain changes may begin years before clinical symptoms become apparent, with overt manifestations usually emerging later in life. This extended latency period provides a valuable opportunity for early detection and prevention. Current treatment strategies for cognitive impairment mainly target symptom control and delayed progression, yet their effectiveness and accessibility are still limited (5). By contrast, prevention strategies targeting modifiable risk factors may have greater public health relevance, particularly in low- and middle-income countries (LMICs), where the burden of cognitive decline is substantial (6). These considerations support a shift toward comprehensive prevention strategies centered on non-pharmacological interventions and modifiable risk factors. Among numerous risk factors, nutritional status has been recognized as a key modifiable factor influencing cognitive function in older adults (7–9). Previous research has shown significant associations between malnutrition and cognitive deterioration (9–11). Importantly, these two conditions may interact, potentially forming a vicious cycle (12). Consequently, international guidelines recommend nutritional evaluation and management as integral components of multi-domain strategies for preventing cognitive decline (12, 13). Identifying individuals at high risk of developing MCI using conventional, easily obtainable indicators at early or preclinical stages remains crucial for clinical and public health practice. Although nutritional assessment tools such as the GNRI (14) and CONUT (15) score are available, they often require serum albumin, lymphocyte counts, ideal body weight, or scoring algorithms. These requirements increase complexity and may limit their use in outpatient follow-up, primary care, and population-level screening (16).

Previous studies have identified triglycerides (TG) as a significant form of energy storage in the body, reflecting nutritional adequacy and caloric reserves to a certain extent (17, 18). Body weight reflects the long-term balance between energy intake and expenditure and may serve as an indirect marker of overall nutritional and energy reserves (19). Total cholesterol (TC), a component of nutritional screening tools such as the CONUT score, is also used as an auxiliary marker of nutritional status (15). Based on these considerations, Doi et al. (16) developed the triglyceride-cholesterol-body weight index (TCBI), an integrated nutritional index based on triglycerides, total cholesterol, and body weight. TCBI showed a moderate but significant correlation with the GNRI, supporting its use as a simplified nutritional assessment tool (16). Moreover, previous studies have linked cognitive decline to TG levels, body weight, and changes in these measures, potentially through vascular function, metabolic homeostasis, inflammation, and nutritional reserves (20–24). Total cholesterol is essential for maintaining cell membrane stability, steroid hormone synthesis, and normal neurological functions (25, 26). Thus, although TCBI is not a cognitive assessment tool, it is simple, easy to calculate, and readily interpretable. These features may make it useful for identifying individuals at high risk of MCI in primary care and community health settings. However, evidence regarding the relationship between TCBI and MCI remains scarce. A recent cross-sectional study found an inverse association between TCBI levels and MCI prevalence among middle-aged and older adults, providing preliminary support (27). However, its cross-sectional nature prevents establishing temporal relationships. Additionally, all-cause mortality may preclude the subsequent occurrence or observation of incident MCI (28). Therefore, using data from the China Health and Retirement Longitudinal Study (CHARLS), we examined the longitudinal association between TCBI and incident MCI in middle-aged and older adults and evaluated the robustness of this association using competing-risk analyses.

## 2 Materials and methods

### 2.1 Study population and design

**Figure 1:**
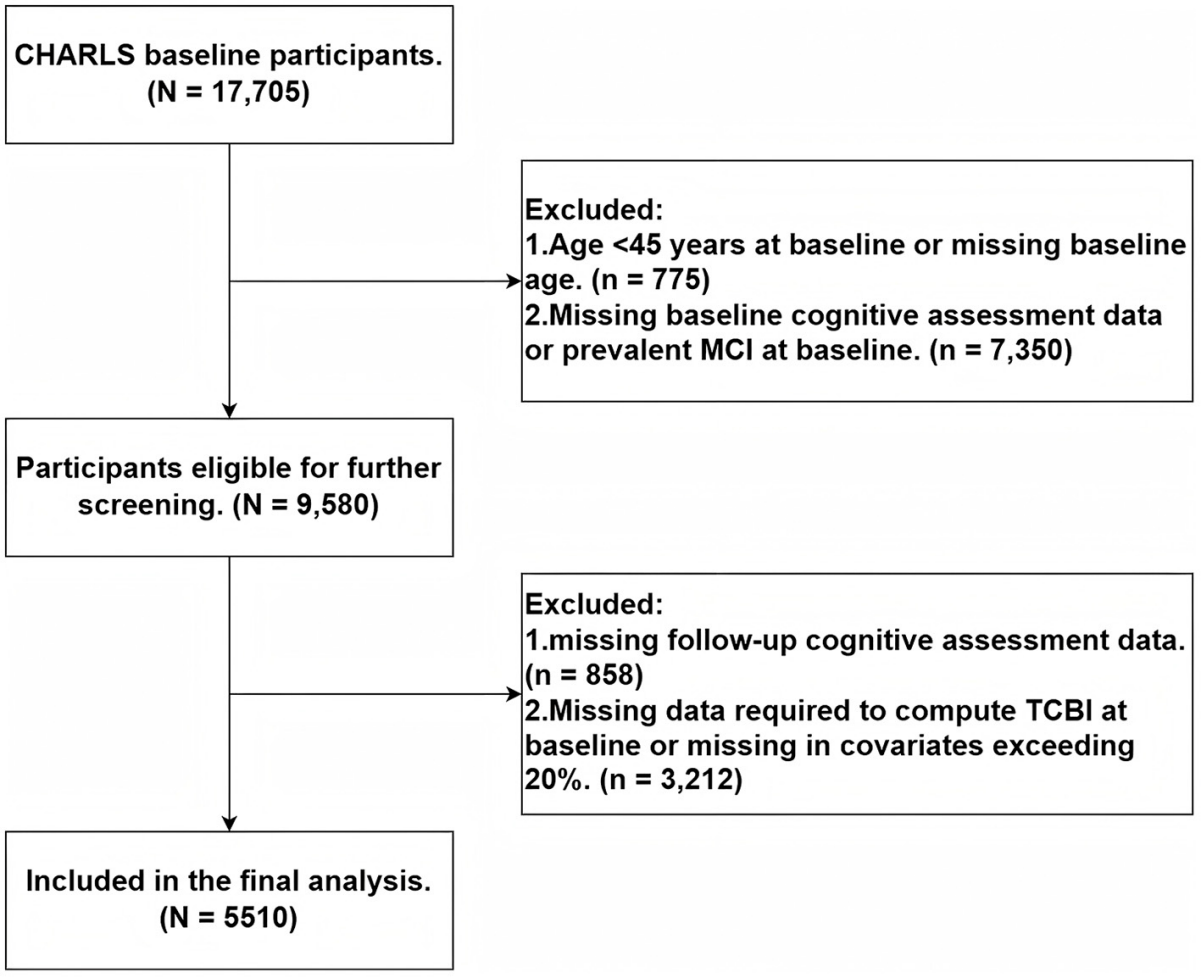
Flowchart of study participant inclusion and exclusion.

This study used data from CHARLS, a nationally representative longitudinal survey conducted from 2011 to 2018. CHARLS was initiated in 2011 and used a multistage stratified sampling design. After the 2011 baseline survey, follow-up assessments were conducted in 2013, 2015, and 2018 to capture changes in health status, socioeconomic conditions, and demographic characteristics among Chinese adults aged 45 years or older (29). The study was approved by the Biomedical Ethics Committee of Peking University (IRB00001052-11015). All procedures adhered to the Declaration of Helsinki, and informed consent was obtained from all participants. Since this was a secondary analysis of publicly available, de-identified data, additional institutional review board approval was not required. From 17,705 participants at baseline in 2011, 12,195 were excluded based on the following criteria: age < 45 years or missing baseline age data (*n* = 775), missing baseline cognitive assessment data or already diagnosed with MCI at baseline (*n* = 7,350), missing cognitive assessment data in follow-up surveys (2013, 2015, or 2018) (*n* = 858), or missing baseline TCBI data or covariate data exceeding a 20% missing rate (*n* = 3,212). After these exclusions, 5,510 eligible participants were included in the final analysis (Figure 1).

### 2.2 Definition of TCBI

Trained medical personnel from the Chinese Center for Disease Control and Prevention (China CDC) collected venous blood samples following standard protocols. The samples were promptly shipped to China CDC's Beijing headquarters, where they were processed and stored at −80 °C. Subsequently, samples were transferred to the Clinical Laboratory Center at Capital Medical University for biochemical analyses. Lipid indicators, including TC, triglycerides (TG), low-density lipoprotein cholesterol (LDL-C), and high-density lipoprotein cholesterol (HDL-C) were measured using enzymatic colorimetric methods. TCBI was calculated according to the formula proposed by Doi et al. (16):


$$\begin{array}{l} \text{TCBI}=\left(\text{TC}\left(\text{mg}/\text{dL}\right)\times \text{T}\text{G}\left(\text{m}\text{g}/\text{d}\text{L}\right)\times \text{B}\text{o}\text{d}\text{y}\;\text{W}\text{e}\text{i}\text{g}\text{h}\text{t}\;\left(\text{k}\text{g}\right)\right)/1,000 \end{array}$$

Because TCBI had a skewed distribution, log10 transformation was applied to reduce skewness and improve model fit, consistent with previous studies (27, 30).

### 2.3 Outcome definition: incident MCI and competing events

The primary outcome was incident MCI during follow-up. CHARLS assessed cognitive function using a standardized questionnaire consisting primarily of the Word Recall Test (WRT) and the Mental Status Test (MST). The WRT evaluated episodic memory; participants listened to 10 words read by the interviewer and were asked to recall them immediately and again after approximately 5 min. One point was awarded per correctly recalled word, resulting in a maximum of 10 points per recall and a total possible score of 20 points. The MST comprised the Telephone Interview for Cognitive Status-10 (TICS-10), which assessed serial subtraction and time orientation, and a drawing test. The maximum MST score was 11 points. The total cognitive score was the sum of WRT and MST scores, with a maximum possible score of 31 points (31). The assessment procedure was consistently repeated during each follow-up survey, with higher scores indicating better cognitive function. We defined MCI based on the Aging-Associated Cognitive Decline (AACD) criteria, drawing upon established approaches from previous studies (32–35). Participants aged 45–59 years constituted one group, while those aged 60 years and above were divided into five-year age bands. Due to the relatively small sample size (*n* = 200) among individuals aged 80 or older, we combined them into a single group for diagnosis, a decision aligned with prior research to ensure stable estimates of means and standard deviations (SD). Participants whose total cognitive scores fell more than one SD below the mean for their respective age group were classified as having MCI. Exact cutoff values are presented in Supplementary Table 1.

Because death during follow-up could preclude subsequent observation of MCI, all-cause mortality was treated as a competing event in sensitivity analyses. Participants who died without developing MCI were classified as competing events. If participants died after developing MCI, they were counted as having incident MCI according to event timing, and death was no longer considered a competing event for these individuals (28, 36, 37).

### 2.4 Covariate assessment

Covariates included in this study comprised sociodemographic factors, behavioral factors, physical health status, and mental health status. Sociodemographic factors included age, sex, education level, marital status, and place of residence (urban/rural), as these may influence participants' access to social services and healthcare. Education level was categorized as no formal education, primary or junior high school, and high school or above. Marital status was classified as married or others (including separated, divorced, widowed, or unmarried). Behavioral factors included social participation, smoking status and alcohol consumption status. CHARLS surveyed 11 types of social activities: interacting with friends; participating in community activity rooms (e.g., mahjong, chess, and cards); dancing or exercising in parks or other public areas; engaging in club activities; attending educational courses or training; assisting non-cohabiting relatives, friends, or neighbors; caring for sick or disabled non-cohabitants; volunteering or charity work; stock trading; internet surfing; and other activities. Social participation was defined as involvement in at least one of these activities in the past month. Smoking status was defined according to lifetime smoking history: never smokers were those who had smoked no more than 100 cigarettes; those who smoked more than 100 cigarettes were categorized as current smokers if currently smoking and ex-smokers if they had ceased smoking (38). Alcohol consumption status was classified as never drinkers (no alcohol consumption in the past year and no previous drinking history), ex-drinkers (no alcohol in the past year but previous drinking history), and current drinkers (alcohol consumption within the past year) (29). Physical health conditions included stroke, diabetes, and hypertension. Participants were classified as having hypertension if they reported a physician diagnosis, current antihypertensive medication use, or exhibited an average systolic blood pressure ≥140 mmHg or DBP ≥90 mmHg based on multiple measurements (39). Diabetes was similarly defined by self-reported diagnosis, use of glucose-lowering medications or insulin, or laboratory findings of fasting glucose ≥126 mg/dL or HbA1c ≥6.5% (26). Other chronic conditions were self-reported by participants (30). Mental health status was assessed using the 10-item Center for Epidemiologic Studies Depression Scale (CESD-10); a score of ≥10 indicated the presence of depressive symptoms, consistent with prior studies (27, 40). Nighttime sleep duration was obtained from self-reported responses to the CHARLS question on average actual nighttime sleep duration during the past month and was included in the multivariable model as a continuous variable in hours. Because TC, TG, and body weight are direct components of TCBI, and because dyslipidemia partly overlaps with TCBI in both conceptual and informational content, these variables were not additionally included in the main multivariable models to avoid overadjustment and potential multicollinearity. A sensitivity analysis was conducted after excluding participants with baseline dyslipidemia, defined by self-reported physician diagnosis, current lipid-modifying therapy, or meeting any of the following criteria: TC ≥240 mg/dL, TG ≥150 mg/dL, LDL-C ≥160 mg/dL, or HDL-C < 40 mg/dL.

### 2.5 Statistical analysis

Continuous variables were summarized according to their distribution and are presented as mean ± SD or median with interquartile range (IQR), as appropriate. In baseline characteristic comparisons, continuous variables were evaluated using the Kruskal-Wallis rank-sum test (non-normally distributed) or one-way analysis of variance (ANOVA) (approximately normally distributed). Categorical variables were compared using Pearson's chi-square test. Cox proportional hazards models were used to estimate the association between Lg TCBI and incident MCI. Results are reported as hazard ratios (HRs) with 95% confidence intervals (CIs). HRs were reported per 1-unit increase in Lg TCBI, per 1 SD increase, and by quartiles, with P for trend calculated accordingly. Three sequentially adjusted models were established based on previous clinical literature (27): Model 1 was unadjusted. Model 2 was adjusted for age and sex. Model 3 was further adjusted for place of residence, education level, marital status, smoking status, alcohol consumption status, social participation, hypertension, diabetes, stroke, cardiac disease, depressive symptoms, diastolic blood pressure (DBP), systolic blood pressure (SBP), LDL-C, HDL-C, fasting plasma glucose, and nighttime sleep duration. Multicollinearity was assessed using variance inflation factors (VIFs). All covariates had VIFs below 5 (Supplementary Table 2). To explore potential non-linear associations between Lg TCBI and MCI risk, restricted cubic spline (RCS) regression was conducted based on Model 3, using the median Lg TCBI as the reference and placing four knots at the 5th, 35th, 65th, and 95th percentiles. The likelihood ratio test was utilized to compare goodness-of-fit between linear and non-linear models. When the RCS suggested a non-linear relationship, a piecewise Cox proportional hazards model was further applied for threshold analysis, with two inflection points identified by iterative search to maximize the log-likelihood, thereby dividing the exposure into three intervals. Goodness-of-fit between linear and piecewise models was compared via likelihood ratio tests, and interval-specific HRs (95% CIs) were reported. Subgroup analyses were conducted to assess consistency across different populations, stratified by age ( ≤ 60, >60 years), sex, place of residence, education level, marital status, smoking status, alcohol consumption status, social participation, diabetes, hypertension, cardiac disease, dyslipidemia, stroke, depressive symptoms, and BMI ( ≤ 24, >24 kg/m^2^). Each subgroup model adjusted for the same covariates as Model 3, except for the stratification variable itself. Sensitivity analyses were performed to verify the robustness of the primary results. First, considering all-cause mortality as a competing event, Fine–Gray subdistribution hazard models (28) were employed to evaluate the association between TCBI and cumulative risk of incident MCI. Definitions and methodological handling of competing risks followed established studies, and results were presented as subdistribution hazard ratios (sHRs) with 95% confidence intervals (CIs) (28, 36, 37). Second, to minimize potential confounding effects from baseline dyslipidemia, multivariable Cox proportional hazards regression analyses were repeated in a subgroup excluding participants with dyslipidemia to reassess the association between TCBI and MCI incidence. A two-sided *P*-value < 0.05 indicated statistical significance. The proportion of missing data was low (maximum 1.379%, Supplementary Table 3). Missing covariates were imputed using the random forest-based non-parametric imputation method (missForest), incorporating exposure variables, follow-up duration, outcome indicators, and all covariates to reduce bias. All statistical analyses were conducted using R software (version 4.2.2, R Foundation for Statistical Computing, Vienna, Austria).

## 3 Results

### 3.1 Baseline characteristics of the study cohort

Baseline demographic, clinical, and biochemical characteristics of participants according to Lg TCBI quartiles are presented in Table 1. A total of 5,510 participants were included, comprising 3,026 males (54.9%) and 2,484 females (45.1%), with an average age of 58 ± 9 years. During the 7-year follow-up, 1,063 incident MCI cases (19.3%) and 437 all-cause deaths (7.9%) occurred. With increasing quartiles of Lg TCBI, significant differences emerged across multiple baseline characteristics. Participants in higher Lg TCBI quartiles were more likely to be female, reside in urban areas, have higher education levels, and engage in social participation (all *P* < 0.05). Regarding behavioral factors, the proportion of current smokers decreased as Lg TCBI increased (*P* < 0.001), whereas alcohol consumption status did not differ significantly across groups (*P* = 0.370). The prevalence of hypertension, diabetes, dyslipidemia, and cardiac disease increased across higher Lg TCBI quartiles. Additionally, systolic blood pressure, DBP, BMI, waist circumference, LDL-C, fasting plasma glucose, and HbA1c increased significantly across quartiles (all *P* < 0.001). Conversely, HDL-C levels declined with higher Lg TCBI (*P* < 0.001). The proportion of participants with depressive symptoms was lower in higher quartiles (*P* < 0.001). There were no significant differences in marital status, stroke prevalence, or nighttime sleep duration among quartile groups (all *P* > 0.05). Regarding follow-up outcomes, higher quartiles of Lg TCBI were associated with significantly lower incidence rates of MCI and all-cause mortality (all *P* < 0.05).

**Table 1:** Baseline characteristics of the study population according to quartiles of Lg TCBI.

Characteristic	Lg TCBI quartiles					*p*-value
	Total (*N* = 5,510)	Q1 (*N* = 1,378)	Q2 (*N* = 1,377)	Q3 (*N* = 1,377)	Q4 (*N* = 1,378)	
Age (years)	58 ± 9	59 ± 10	58 ± 9	58 ± 9	57 ± 8	< 0.001
Sex						< 0.001
Male	3,026 (54.9%)	831 (60.3%)	781 (56.7%)	705 (51.2%)	709 (51.5%)	
Female	2,484 (45.1%)	547 (39.7%)	596 (43.3%)	672 (48.8%)	669 (48.5%)	
Place of residence						< 0.001
Urban	2,244 (40.7%)	456 (33.1%)	541 (39.3%)	580 (42.1%)	667 (48.4%)	
Rural	3,266 (59.3%)	922 (66.9%)	836 (60.7%)	797 (57.9%)	711 (51.6%)	
Education level						0.021
No formal education	1,717 (31.2%)	466 (33.8%)	418 (30.4%)	435 (31.6%)	398 (28.9%)	
Primary/middle school	2,987 (54.2%)	745 (54.1%)	748 (54.3%)	726 (52.7%)	768 (55.7%)	
High school or above	806 (14.6%)	167 (12.1%)	211 (15.3%)	216 (15.7%)	212 (15.4%)	
Marital status						0.169
Married	4,963 (90.1%)	1,225 (88.9%)	1,235 (89.7%)	1,244 (90.3%)	1,259 (91.4%)	
Other	547 (9.9%)	153 (11.1%)	142 (10.3%)	133 (9.7%)	119 (8.6%)	
Smoking status						< 0.001
Current smoker	1,870 (33.9%)	549 (39.8%)	493 (35.8%)	415 (30.1%)	413 (30.0%)	
Former smoker	580 (10.5%)	141 (10.2%)	126 (9.2%)	147 (10.7%)	166 (12.0%)	
Never smoker	3,060 (55.5%)	688 (49.9%)	758 (55.0%)	815 (59.2%)	799 (58.0%)	
Alcohol consumption status						0.370
Current drinker	2,028 (36.8%)	535 (38.8%)	520 (37.8%)	488 (35.4%)	485 (35.2%)	
Former drinker	479 (8.7%)	124 (9.0%)	116 (8.4%)	121 (8.8%)	118 (8.6%)	
Never drinker	3,003 (54.5%)	719 (52.2%)	741 (53.8%)	768 (55.8%)	775 (56.2%)	
Social participation	3,118 (56.6%)	706 (51.2%)	776 (56.4%)	805 (58.5%)	831 (60.3%)	< 0.001
Cardiac disease	665 (12.1%)	137 (9.9%)	151 (11.0%)	175 (12.7%)	202 (14.7%)	< 0.001
Hypertension	2,240 (40.7%)	389 (28.2%)	498 (36.2%)	617 (44.8%)	736 (53.4%)	< 0.001
Stroke	100 (1.8%)	21 (1.5%)	19 (1.4%)	31 (2.3%)	29 (2.1%)	0.237
Dyslipidemia	2,763 (50.1%)	210 (15.2%)	440 (32.0%)	786 (57.1%)	1,327 (96.3%)	< 0.001
Diabetes	947 (17.2%)	143 (10.4%)	179 (13.0%)	240 (17.4%)	385 (27.9%)	< 0.001
Depressive symptoms	1,711 (31.1%)	481 (34.9%)	438 (31.8%)	416 (30.2%)	376 (27.3%)	< 0.001
Antihyperlipidemic agents	331 (6.0%)	39 (2.8%)	71 (5.2%)	82 (6.0%)	139 (10.1%)	< 0.001
Antihypertensive agents	1,126 (20.4%)	150 (10.9%)	249 (18.1%)	315 (22.9%)	412 (29.9%)	< 0.001
SBP (mmHg)	130 ± 21	126 ± 21	129 ± 20	131 ± 21	135 ± 21	< 0.001
DBP (mmHg)	76 ± 12	73 ± 12	75 ± 12	77 ± 12	80 ± 12	< 0.001
BMI (kg/m^2^)	23.8 ± 3.9	21.6 ± 2.9	23.0 ± 3.4	24.6 ± 3.6	26.2 ± 4.0	< 0.001
Body weight (kg)	61 ± 11	54 ± 9	59 ± 9	63 ± 11	68 ± 12	< 0.001
Body height (cm)	160 ± 8	159 ± 8	160 ± 8	160 ± 8	161 ± 8	< 0.001
WC (cm)	85 ± 12	79 ± 11	83 ± 11	87 ± 12	91 ± 12	< 0.001
TG (mg/dL)	107 (76, 158)	63 (53, 73)	92 (79, 105)	127 (109, 147)	212 (171, 285)	< 0.001
TC (mg/dL)	194 ± 38	169 ± 29	187 ± 31	200 ± 32	219 ± 40	< 0.001
HDL-C (mg/dL)	50 ± 15	58 ± 15	54 ± 15	48 ± 13	41 ± 12	< 0.001
LDL-C (mg/dL)	117 ± 35	100 ± 25	117 ± 29	127 ± 32	123 ± 45	< 0.001
FPG (mg/dL)	103 (95, 114)	99 (91, 107)	101 (94, 110)	103 (95, 113)	109 (100, 126)	< 0.001
HbA1c (%)	5.10 (4.90, 5.40)	5.00 (4.80, 5.30)	5.10 (4.80, 5.40)	5.10 (4.90, 5.40)	5.20 (5.00, 5.60)	< 0.001
Nighttime sleep duration (hours)	6.44 ± 1.72	6.40 ± 1.74	6.48 ± 1.73	6.38 ± 1.70	6.51 ± 1.69	0.147
TCBI	3.11 ± 0.31	2.74 ± 0.13	2.99 ± 0.06	3.19 ± 0.06	3.52 ± 0.20	< 0.001
Death	437 (7.9%)	134 (9.7%)	108 (7.8%)	97 (7.0%)	98 (7.1%)	0.032
MCI	1,063 (19.3%)	306 (22.2%)	290 (21.1%)	242 (17.6%)	225 (16.3%)	< 0.001

SBP, systolic blood pressure; DBP, diastolic blood pressure; BMI, body mass index; WC, waist circumference; TG, triglycerides; TC, total cholesterol; HDL-C, high-density lipoprotein cholesterol; LDL-C, low-density lipoprotein cholesterol; FPG, fasting plasma glucose; HbA1c, glycated hemoglobin; TCBI, triglyceride-cholesterol-body weight index (log10-transformed); MCI, mild cognitive impairment.

### 3.2 Association between Lg TCBI and incident MCI

**Figure 2:**
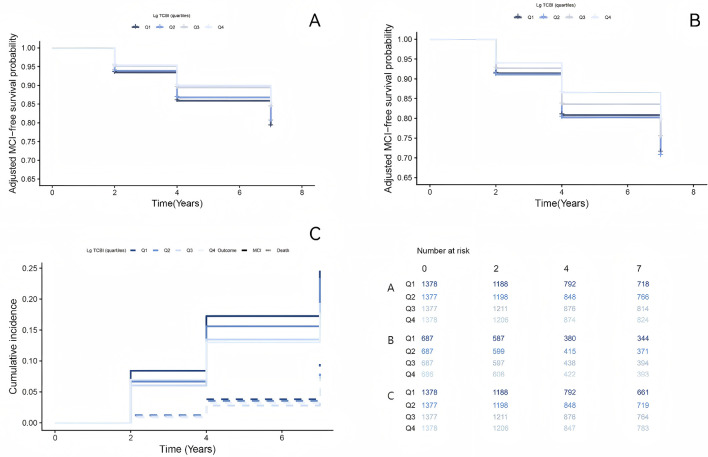
Association between TCBI and incident MCI based on multivariable-adjusted survival models. **(A)** Multivariable-adjusted MCI-free survival curves from the Cox proportional hazards model. **(B)** Multivariable-adjusted MCI-free survival curves from the Cox proportional hazards model among participants without baseline dyslipidemia. **(C)** Cumulative incidence function (CIF) curves for incident MCI derived from the Fine–Gray subdistribution hazard model with all-cause mortality as a competing event, with the cumulative incidence curve for all-cause mortality shown simultaneously. Adjusted for age, sex, place of residence, education level, marital status, smoking status, alcohol consumption status, social participation, hypertension, diabetes, stroke, cardiac disease, depressive symptoms, diastolic blood pressure (DBP), systolic blood pressure (SBP), LDL-C, HDL-C, fasting plasma glucose, and nighttime sleep duration.

Results of the Cox regression analysis examining associations between Lg TCBI and incident MCI are presented in Table 2 and Figure 2A. During the 7-year follow-up, 1,063 incident MCI cases occurred. When modeled as a continuous variable, each 1-unit increase in Lg TCBI was associated with a lower risk of MCI. This association persisted after full covariate adjustment in Model 3 (HR = 0.73, 95% CI 0.57–0.94, *P* = 0.014). Similarly, when standardized per 1 SD increase, higher Lg TCBI remained significantly associated with reduced MCI risk (Model 3: HR = 0.91, 95% CI 0.84–0.98, *P* = 0.014). In quartile analyses, participants in Q3 (HR = 0.76, 95% CI 0.63–0.92, *P* = 0.005) and Q4 (HR = 0.75, 95% CI 0.61–0.93, *P* = 0.007) exhibited significantly lower MCI risk compared to the reference group (Q1). A significant trend across Lg TCBI quartiles was observed (Model 3: *P* for trend = 0.001). However, no significant difference was observed between Q2 and Q1 groups. Overall, higher Lg TCBI levels were consistently associated with lower risks of incident MCI across all adjustment models.

**Table 2:** Association between TCBI and incident MCI: Cox proportional hazards models.

Characteristic	Model 1			Model 2			Model 3		
	HR	95% CI	*p*-value	HR	95% CI	*p*-value	HR	95% CI	*p*-value
TCBI (continuous)	0.66	0.54, 0.80	< 0.001	0.64	0.53, 0.79	< 0.001	0.73	0.57, 0.94	0.014
TCBI (standardized)	0.88	0.82, 0.93	< 0.001	0.87	0.82, 0.93	< 0.001	0.91	0.84, 0.98	0.014
TCBI									
Q1 (1.43, 2.89)	—	—		—	—		—	—	
Q2 (2.89, 3.09)	0.93	0.79, 1.09	0.350	0.92	0.79, 1.08	0.326	0.98	0.83, 1.15	0.780
Q3 (3.09, 3.31)	0.76	0.64, 0.90	0.001	0.74	0.62, 0.87	< 0.001	0.76	0.63, 0.92	0.005
Q4 (3.31, 4.83)	0.70	0.59, 0.84	< 0.001	0.69	0.58, 0.82	< 0.001	0.75	0.61, 0.93	0.007
*P* for trend			< 0.001			< 0.001			0.001

CI, confidence interval; HR, hazard ratio; MCI, mild cognitive impairment; TCBI, triglyceride-cholesterol-body weight index. TCBI was log10-transformed before analysis.

Model 1: Unadjusted.

Model 2: Adjusted for age and sex.

Model 3: Further adjusted for marital status, education level, place of residence, smoking status, alcohol consumption status, social participation, nighttime sleep duration, hypertension, diabetes, stroke, cardiac disease, depressive symptoms, systolic blood pressure (SBP), diastolic blood pressure (DBP), fasting plasma glucose (mg/dL), HDL-C (mg/dL), and LDL-C (mg/dL), based on Model 2.

### 3.3 Dose–Response and non-linear relationship between Lg TCBI and incident MCI

**Figure 3:**
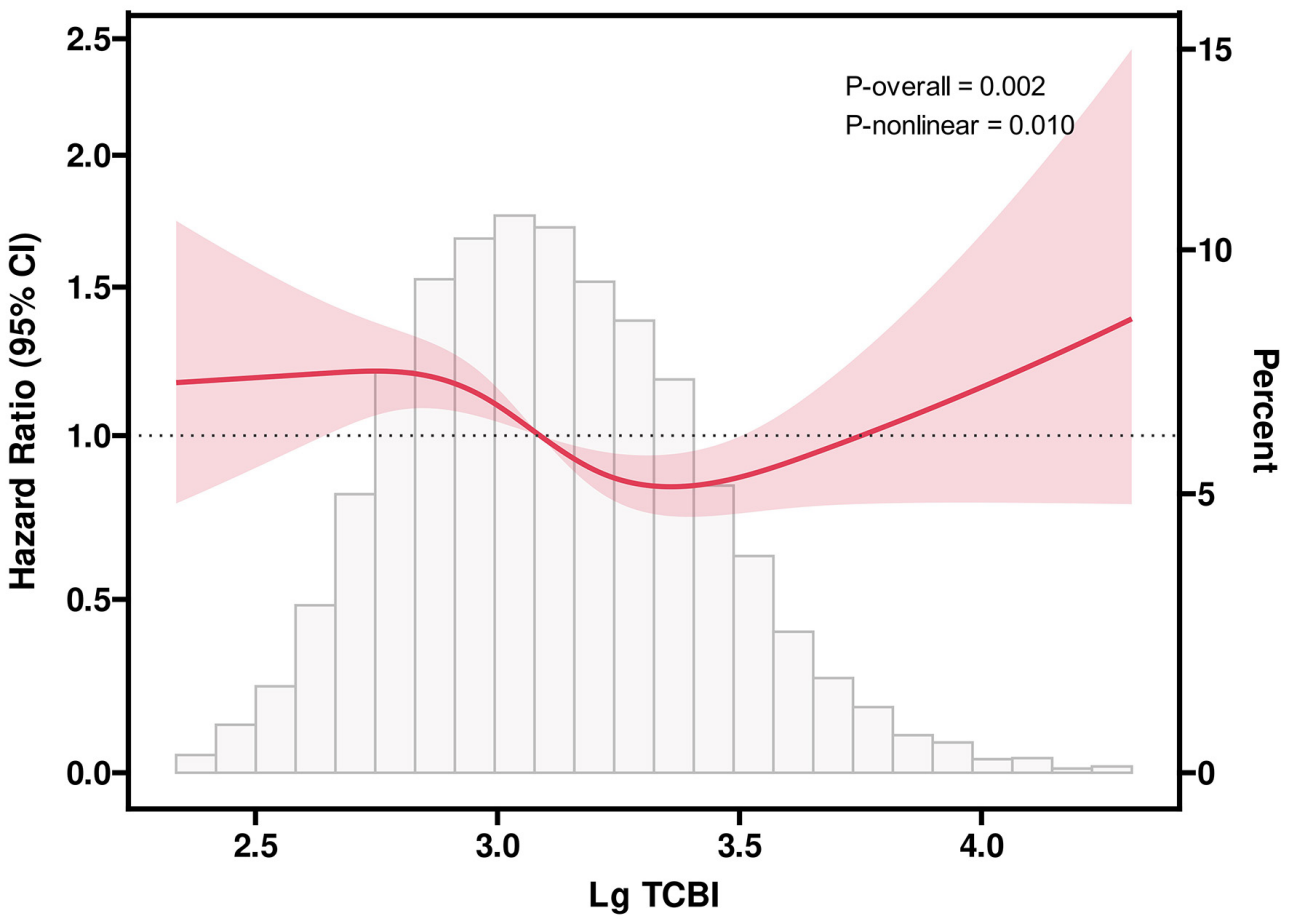
Restricted cubic spline (RCS) analysis showing the non-linear association between TCBI and the risk of incident mild cognitive impairment (MCI). Adjusted for age, sex, place of residence, education level, marital status, smoking status, alcohol consumption status, social participation, hypertension, diabetes, stroke, cardiac disease, depressive symptoms, diastolic blood pressure (DBP), systolic blood pressure (SBP), LDL-C, HDL-C, fasting plasma glucose, and nighttime sleep duration.

Under the covariate-adjustment framework of Model 3, a restricted cubic spline (RCS) analysis was performed to assess the potential non-linear relationship between Lg TCBI and incident MCI risk (Figure 3). The results indicated a significant non-linear association (*P* for nonlinearity < 0.05). Based on these findings, a piecewise Cox proportional hazards model was constructed to examine threshold effects (Table 3). Two inflection points (Lg TCBI = 2.84 and 3.39) were identified by maximizing the log-likelihood. Within the interval 2.84 ≤ Lg TCBI < 3.39, each 1-unit increase in Lg TCBI was significantly associated with a reduced risk of MCI (HR = 0.46, 95% CI: 0.31–0.70, *P* < 0.001). However, no significant associations were observed for intervals Lg TCBI < 2.84 (HR = 1.04, 95% CI: 0.45–2.41, *P* = 0.928) and Lg TCBI ≥3.39 (HR = 1.92, 95% CI: 0.98–3.79, *P* = 0.059). The likelihood ratio test indicated that the piecewise model fit the data better than the linear model (*P* = 0.013), suggesting a possible threshold-dependent or interval-dependent association.

**Table 3:** Threshold effect analysis of TCBI on incident MCI.

Model/TCBI interval	N (Events)	HR (95% CI)	*P*-value
Standard Cox regression model	5510 (1063)	0.73 (0.57, 0.94)	0.014
Piecewise Cox regression model (breakpoints = 2.84, 3.39)			
TCBI < 2.84	254 (55)	1.04 (0.45, 2.41)	0.928
2.84 ≤ TCBI < 3.39	4,896 (944)	0.46 (0.31, 0.70)	< 0.001
TCBI ≥ 3.39	360 (64)	1.92 (0.98, 3.79)	0.059
Log-likelihood ratio test			0.013

CI, confidence interval; HR, hazard ratio; TCBI, triglyceride-cholesterol-body weight index. TCBI was log10-transformed before analysis; MCI, mild cognitive impairment. Log-likelihood ratio represents the likelihood ratio test comparing the standard Cox model with the piecewise Cox regression model. N, total number of participants; Events, number of incident MCI cases.

The model was adjusted for age, sex, marital status, education level, place of residence, smoking status, alcohol consumption status, social participation, nighttime sleep duration, hypertension, diabetes, stroke, cardiac disease, depressive symptoms, systolic blood pressure (SBP), diastolic blood pressure (DBP), fasting plasma glucose (mg/dL), HDL-C (mg/dL), and LDL-C (mg/dL).

### 3.4 Subgroup and interaction analyses

**Figure 4:**
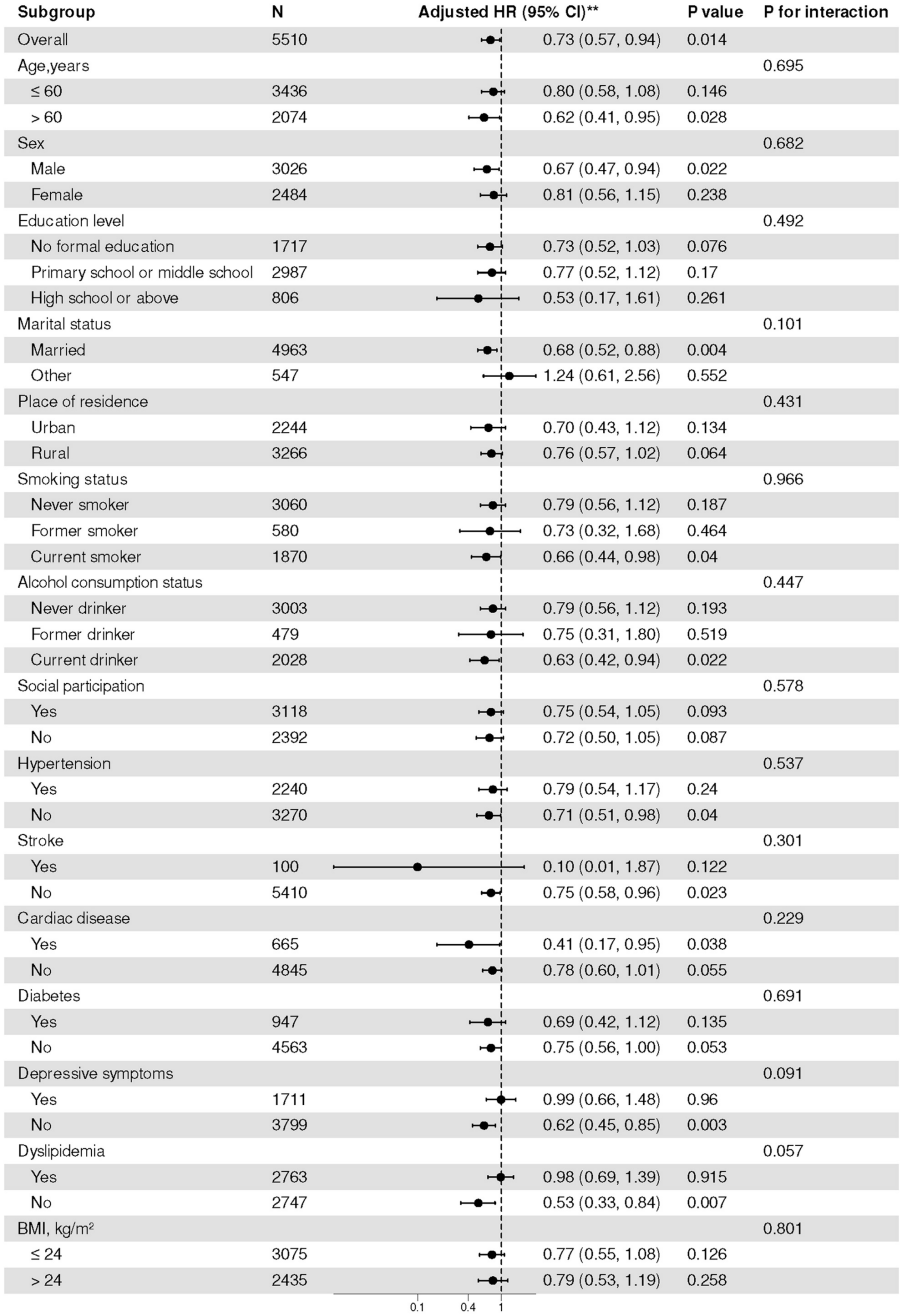
Subgroup analyses of the association between TCBI and the risk of incident mild cognitive impairment (MCI).

The results of subgroup analyses are presented in Figure 4. In the fully adjusted model (adjusted for covariates consistent with Model 3, excluding the stratification variable), Lg TCBI was negatively associated with incident MCI risk in the overall cohort (HR = 0.73, 95% CI: 0.57–0.94, *P* = 0.014). The direction of association was generally consistent across subgroups. Statistically significant associations were found among participants aged >60 years, male, married, current smokers, current drinkers, individuals without hypertension, without stroke, with cardiac disease, without depressive symptoms, and without dyslipidemia. However, the associations in other subgroups did not reach statistical significance. Estimates in some subgroups, such as participants with stroke and those with non-married marital status, were less stable because of small sample sizes and wide confidence intervals. Interaction tests revealed no significant modification effects across stratification variables (all *P* for interaction >0.05), although the interaction by dyslipidemia status approached borderline significance (*P* for interaction = 0.057). Overall, the evidence did not support significant effect modification.

### 3.5 Sensitivity and competing risk analyses

A sensitivity analysis was performed among participants without baseline dyslipidemia (*n* = 2,747; incident MCI cases = 579) using multivariable Cox proportional hazards models (Table 4 and Figure 2B). When treated as a continuous variable, Lg TCBI exhibited a negative association with incident MCI risk in both unadjusted and sequentially adjusted models. In the fully adjusted model (Model 3), each 1-unit increase in Lg TCBI was associated with significantly reduced MCI risk (HR = 0.53, 95% CI: 0.33–0.84, *P* = 0.007). After standardization per 1 SD increase, Lg TCBI remained significantly associated with decreased MCI risk (Model 3: HR = 0.88, 95% CI: 0.80–0.97, *P* = 0.007). Quartile-based analyses showed that participants in Q4 had significantly lower MCI risk compared with Q1 (Model 3: HR = 0.72, 95% CI: 0.55–0.95, *P* = 0.019), while Q2 and Q3 showed no significant differences. The trend test confirmed a statistically significant negative linear trend between Lg TCBI quartiles and MCI risk (Model 3: *P* for trend = 0.023). The observed associations in participants with normal blood lipids aligned consistently with those of the main analysis.

**Table 4:** Association of TCBI with incident MCI among participants without baseline dyslipidemia.

Characteristic	Model 1			Model 2			Model 3		
	HR	95% CI	*p*-value	HR	95% CI	*p*-value	HR	95% CI	*p*-value
TCBI (continuous)	0.52	0.35, 0.77	0.001	0.52	0.35, 0.78	0.001	0.53	0.33, 0.84	0.007
TCBI (per 1-SD increase)	0.88	0.81, 0.95	0.001	0.88	0.81, 0.95	0.001	0.88	0.80, 0.97	0.007
TCBI									
Q1 [1.43,2.79)	—	—		—	—		—	—	
Q2 [2.79,2.93)	1.04	0.84, 1.30	0.706	1.04	0.84, 1.30	0.697	1.04	0.83, 1.31	0.721
Q3 [2.93,3.08)	0.93	0.74, 1.17	0.527	0.93	0.74, 1.17	0.551	0.99	0.78, 1.26	0.926
Q4 [3.08,3.51]	0.70	0.55, 0.89	0.004	0.70	0.55, 0.89	0.004	0.72	0.55, 0.95	0.019
*P* for trend			0.003			0.003			0.023

CI, confidence interval; HR, hazard ratio; MCI, mild cognitive impairment; TCBI, triglyceride-cholesterol-body weight index. TCBI was log10-transformed before analysis.

Model 1: Unadjusted.

Model 2: Adjusted for age and sex.

Model 3: Further adjusted for marital status, education level, place of residence, smoking status, alcohol consumption status, social participation, nighttime sleep duration, hypertension, diabetes, stroke, cardiac disease, depressive symptoms, systolic blood pressure (SBP), diastolic blood pressure (DBP), fasting plasma glucose (mg/dL), HDL-C (mg/dL), and LDL-C (mg/dL), based on Model 2.

We evaluated the potential for differential loss to follow-up. Compared with the remaining participants, those lost to follow-up who did not develop MCI or die prior to their loss differed slightly in age (SMD = 0.23) and education level (SMD = 0.33), indicating selective attrition based on sociodemographic factors. However, differences in baseline Lg TCBI and most metabolic indicators were small (SMD < 0.2). Further Cox regression analyses, with loss to follow-up as the outcome, revealed no significant association between baseline Lg TCBI and the risk of attrition (all *P* > 0.05). This suggests that the primary findings of this study were unlikely to be influenced by informative censoring (Supplementary Table 4). During the 7-year follow-up, 1,063 incident MCI cases (19.29%) and 437 all-cause mortality cases (7.93%) occurred. Considering that mortality could preclude subsequent MCI observation, we utilized the Fine–Gray subdistribution hazard model, defining all-cause mortality as a competing event (Table 5 and Figure 2C). In the unadjusted model, compared to the lowest Lg TCBI quartile (Q1), higher quartiles were associated with significantly reduced subdistribution hazards (sHR) for incident MCI, specifically Q3 (sHR = 0.77, 95% CI: 0.65–0.90, *P* = 0.001) and Q4 (sHR = 0.72, 95% CI: 0.61–0.85, *P* < 0.001). However, Q2 did not significantly differ from Q1 (sHR = 0.93, 95% CI: 0.80–1.08, *P* = 0.359). After multivariable adjustment, the pattern remained similar: compared to Q1, participants in Q3 (sHR = 0.78, 95% CI: 0.65–0.93, *P* = 0.007) and Q4 (sHR = 0.77, 95% CI: 0.63–0.94, *P* = 0.010) exhibited significantly lower subdistribution hazards for incident MCI, whereas Q2 remained nonsignificant (sHR = 0.96, 95% CI: 0.82–1.12, *P* = 0.569). The quartile-based trend test remained statistically significant in the fully adjusted model (P for trend = 0.002). When analyzing Lg TCBI continuously or per 1-SD increase, results were directionally consistent. In the fully adjusted model, each 1-unit increase in Lg TCBI was associated with a reduced subdistribution hazard for incident MCI (sHR = 0.75, 95% CI: 0.59–0.96); similarly, each 1-SD increase corresponded to a lower subdistribution hazard (sHR = 0.91, 95% CI: 0.85–0.99). Overall, higher Lg TCBI levels remained associated with reduced risk of incident MCI after accounting for the competing risk of death.

**Table 5:** Association of TCBI with incident mild cognitive impairment (MCI): Fine–Gray subdistribution hazard model with all-cause mortality as a competing event.

Characteristic	Model 1			Model 2			Model 3		
	sHR	95% CI	*p*-value	sHR	95% CI	*p*-value	sHR	95% CI	*p*-value
TCBI (continuous)	0.67	0.55, 0.82	< 0.001	0.66	0.54, 0.80	< 0.001	0.75	0.59, 0.96	0.021
TCBI (per 1-SD increase)	0.88	0.83, 0.94	< 0.001	0.88	0.83, 0.93	< 0.001	0.91	0.85, 0.99	0.021
TCBI									
Q1 [1.43,2.89)	—	—		—	—		—	—	
Q2 [2.89,3.09)	0.93	0.80, 1.08	0.359	0.93	0.79, 1.08	0.322	0.96	0.82, 1.12	0.569
Q3 [3.09,3.31)	0.77	0.65, 0.90	0.001	0.75	0.64, 0.88	< 0.001	0.78	0.65, 0.93	0.007
Q4 [3.31,4.83]	0.72	0.61, 0.85	< 0.001	0.70	0.60, 0.83	< 0.001	0.77	0.63, 0.94	0.010
*P* for trend			< 0.001			< 0.001			0.002

CI, confidence interval; sHR, subdistribution hazard ratio; MCI, mild cognitive impairment; TCBI, triglyceride-cholesterol-body weight index. TCBI was log10-transformed prior to analysis.

Model 1: Unadjusted.

Model 2: Adjusted for age and sex.

Model 3: Additionally adjusted for marital status, education level, place of residence, smoking status, alcohol consumption status, social participation, nighttime sleep duration, hypertension, diabetes, stroke, cardiac disease, depressive symptoms, systolic blood pressure (SBP), diastolic blood pressure (DBP), fasting plasma glucose (mg/dL), HDL-C (mg/dL), and LDL-C (mg/dL).

## 4 Discussion

Using nationally representative CHARLS cohort data, this study examined the longitudinal association between TCBI, a novel nutritional index, and incident MCI. During a 7-year follow-up of 5,510 middle-aged and older Chinese adults, higher TCBI was significantly associated with a reduced risk of MCI after adjusting for demographic, lifestyle, and clinical metabolic factors. This association was also observed in Fine–Gray competing-risk analyses that treated all-cause mortality as a competing event and in analyses restricted to participants without baseline dyslipidemia. In subgroup analyses, the direction of the effect estimates was generally consistent with the overall findings, and interaction tests revealed no significant evidence of effect modification. Although the interaction by dyslipidemia status was of borderline significance (*P* for interaction = 0.057), all other interactions were nonsignificant. The phenomenon of significant associations observed in some subgroups but not others may be attributable to statistical variability resulting from differences in subgroup sample sizes, event numbers, and estimation precision, rather than reflecting true inter-group differences (41). This effect was particularly evident in the subgroup with stroke.

This study extends and deepens the cross-sectional findings reported by Liu et al. (27), who first observed a negative association between TCBI and MCI. The longitudinal design preserves the temporal ordering between exposure and outcome, thereby reducing the likelihood of reverse causation, although it cannot be completely eliminated. Furthermore, given that death among older adults can preclude subsequent observation of MCI occurrence, we conducted competing risk sensitivity analyses with all-cause mortality. The results confirmed that the inverse association between TCBI and MCI persisted after accounting for the competing risk of death, alleviating concerns about potential biases arising from competing events.

TCBI has also been linked to clinical outcomes in patients with stroke (42), acute coronary syndrome (43), and sarcopenia (44), suggesting potential clinical utility. Compared with individual lipid markers, TCBI integrates lipid metabolism with weight-related nutritional characteristics and may therefore better reflect overall metabolic status and nutritional reserves.

Our findings are consistent with previous evidence linking nutritional status to cognitive function. Given the unique energy metabolic demands of the brain, cognitive decline depends not only on the degeneration of brain structures and functions but also on adequate nutrient intake and nutritional reserves (45, 46). Several clinical studies have reported that malnutrition is associated with a higher risk of cognitive impairment (10, 47, 48), and that weight loss and dietary changes may precede the onset of cognitive impairment (49). These observations align with our results, suggesting a potential temporal relationship between nutritional changes and cognitive decline. However, it is also important to consider that CI may directly diminish an individual's ability to purchase food, prepare meals, and maintain regular eating habits, resulting in simplified and monotonous dietary patterns that elevate malnutrition risk (50). Furthermore, cognitive decline may exacerbate age-related dysphagia and eating difficulties (50, 51), thereby reinforcing a vicious cycle between cognitive deterioration and malnutrition (12). Therefore, early identification and management of nutritional risk in individuals with cognitive decline may help interrupt this cycle and delay functional deterioration. Current studies suggest dietary patterns such as the Mediterranean diet and Mediterranean-DASH Intervention for Neurodegenerative Delay (MIND) diet may be associated with reduced risk of age-related CI, including cognitive decline, dementia, and Alzheimer's disease (52–54); however, findings across different studies remain inconsistent (55). Some dietary intervention trials have not demonstrated improvements in global cognitive or neuroimaging outcomes, and evidence regarding nutritional supplement interventions remains limited and inconclusive. Thus, further high-quality studies are needed to confirm these findings (5, 56). Nonetheless, existing guidelines and expert consensus continue to advocate nutritional improvement, as it is considered to provide potential benefits for cognitive health.

The relationship between nutritional status and cognitive function, however, remains complex and incompletely understood. For example, Schüler et al. (57) indicated that overnutrition can also impair cognitive function. A recent review summarized that this relationship is not entirely mediated by weight gain alone. Even short-term exposure to overnutrition, before obesity develops, can induce brain insulin resistance (IR), alter gut microbiota, increase intestinal barrier permeability, promote the entry of endotoxins and microbial-related products into circulation, and subsequently affect the central nervous system via the gut–brain axis (58). These metabolic and immune abnormalities collectively activate inflammatory mediators, damaging tight junctions and increasing blood-brain barrier permeability, thus exacerbating neuroinflammation and cognitive decline.

Notably, our findings suggest that the association between TCBI and MCI risk may not be strictly linear. In the Cox models using quartiles, we observed no significant difference between Q1 and Q2, possibly because their TCBI levels were relatively close. This indicates that relying solely on categorical grouping or linear assumptions may be insufficient to accurately characterize the true relationship. Restricted cubic spline and threshold analyses suggested a nonlinear, interval-dependent association. Specifically, the inverse association was most apparent within the intermediate range of TCBI, whereas associations at the lower and higher ends of the distribution were not statistically significant. Considering the distribution of exposure values, the relatively small sample sizes at the lower and upper extremes limited the precise estimation of threshold locations and effect sizes. Consequently, we interpret these findings as indicative evidence supporting a non-linear or interval-dependent relationship, rather than confirmation of a definitive threshold.

Although TCBI provides an overall measure of nutritional and metabolic status, its individual components, particularly peripheral TC and TG, do not directly correspond to their intracerebral counterparts. Although cholesterol is essential for normal neuronal function, peripheral cholesterol is transported mainly in lipoprotein particles and has limited ability to cross the blood–brain barrier (BBB). According to Victor et al. (59) brain cholesterol is primarily synthesized locally. Thus, serum TC in TCBI likely reflects systemic lipid homeostasis or the general metabolic environment rather than directly representing cerebral cholesterol availability. Furthermore, this relationship changes with age. Recent cohort studies indicate that the relationship between peripheral cholesterol and cognitive outcomes is influenced by age and timing of measurements. Higher levels of TC, LDL-C, and TG in midlife are associated with increased risk of long-term cognitive decline or dementia. However, lower or decreasing cholesterol levels in older adults should not be simply interpreted as protective (60–62). Interestingly, Aine et al. (63) found that higher TC levels were positively associated with performance on verbal memory tasks. Consequently, some researchers propose that low TC in older adults may itself pose risks, potentially impairing brain structure and cognitive function. This consideration is crucial, as neuronal cells are rich in lipids and highly dependent on active oxidative metabolism, making the brain particularly susceptible to oxidative stress. Moreover, ApoE4 can alter cerebral lipid transport and amyloid-beta (Aβ) clearance. Elevated peripheral cholesterol may enter the brain as 27-hydroxycholesterol (27-OHC), exacerbating Aβ pathology. Thus, high cholesterol might amplify cognitive risk in the presence of ApoE4 (64), although this effect modification is inconsistent across population studies (65). Elevated peripheral cholesterol can promote the formation of oxysterols, such as 27-OHC, which cross the BBB, creating a potential mechanistic link between peripheral cholesterol levels and intracerebral lipid homeostasis or neuropathological changes (65, 66). Additionally, elevated peripheral cholesterol may impair cerebral perfusion by promoting atherosclerosis, thereby contributing to CI development and progression. Circulating TG can be hydrolyzed by lipoprotein lipase (LPL) at the cerebral microvascular endothelium, generating free fatty acids (FFAs) and other metabolites capable of crossing into brain tissue. These metabolites may serve as precursors for cerebral energy supply and essential lipid synthesis under certain physiological conditions (67). This pathway may link peripheral energy reserves with cerebral metabolic demands. Evidence from lipidomics studies of synaptic function also points to the importance of lipid homeostasis in maintaining normal synaptic activity (59). Elevated levels of FFAs and TG can disrupt N-methyl-D-aspartate (NMDA) receptor balance within the hippocampus, potentially mediating cognitive deficits (68, 69). Zhou et al. (70) observed better cognitive function among individuals with TG levels within the normal to high-normal range. However, other studies have reported inconsistent results, indicating either negative (71), positive (72, 73) or no correlations (74) between TG and CI. Moreover, evidence suggests that the prognostic relevance of TG on cognitive outcomes may differ depending on the life stage at measurement, early life, middle age, or late life (20, 75). Thus, additional research on TG and TC under varying conditions and across diverse populations is warranted.

Because TCBI is derived from TG, TC, and body weight, it overlaps partly with insulin-resistance surrogate indices such as METS-IR and TyG. Thus, beyond capturing nutritional reserves and lipid metabolism, TCBI might also partly represent insulin resistance-related metabolic states. Consequently, its effect on cognitive function likely involves multiple biological pathways rather than a single mechanism.

## 5 Limitations and future directions

Several limitations of this study should be acknowledged. First, constraints of the CHARLS database prevented full adjustment for potential confounding factors, including detailed dietary patterns and specific medication use. Second, the definition of MCI relied on cognitive measures and operational criteria available in CHARLS, which could introduce measurement errors and misclassification. Third, the findings were derived from a Chinese middle-aged and older population and may not be directly generalizable to other ethnic or cultural settings.

Considering these limitations, future research could proceed in several directions. First, validation in independent cohorts from different countries, ethnic groups, and cultural backgrounds is needed to strengthen external validity. Secondly, future studies should incorporate detailed dietary information, medication history, and longitudinal body-weight changes to reduce residual confounding and clarify underlying mechanisms. Thirdly, employing more systematic clinical or neuropsychological criteria to ascertain MCI would reduce outcome misclassification and enhance the reliability of results. Additionally, combining direct metabolic evaluations, such as hyperinsulinemic-euglycemic clamp tests, could further elucidate the underlying pathways involving TCBI.

## 6 Public health implications

From a public health perspective, TCBI relies only on routine lipid measurements and body weight and does not require complex scales, imaging, or specialized laboratory tests. This makes it practical and potentially scalable, particularly in resource-limited settings. As an easily calculated composite index derived from routine biochemical and anthropometric data, TCBI may support population-level risk stratification. It may also help identify individuals at higher risk of MCI and may be particularly useful in community health management and primary care. Importantly, TCBI should be used only for risk screening and stratification, not as a substitute for formal cognitive assessment or clinical diagnosis of MCI. Individuals with low TCBI values may warrant further nutritional assessment, comprehensive chronic disease management, and regular cognitive follow-up.

## 7 Conclusion

In conclusion, this nationally representative CHARLS cohort study provides evidence of a longitudinal association between TCBI and incident MCI. During 7 years of follow-up, higher TCBI was associated with a lower risk of MCI. This association was also observed after accounting for all-cause mortality as a competing event and after excluding participants with baseline dyslipidemia. Restricted cubic spline and piecewise Cox analyses further suggested a nonlinear, interval-dependent association, with the inverse association most apparent within a moderate TCBI range. Because TCBI is an easily calculated composite index based on routine biochemical markers and body weight, it may have value for population-level risk stratification and identification of high-risk individuals, particularly in community and primary care settings.

## Data Availability

The datasets analyzed for this study can be obtained from the CHARLS database (http://charls.pku.edu.cn/).

## References

[1] Beard JR Officer A de CIA Sadana R Pot AM Michel J . The World report on ageing and health: a policy framework for healthy ageing. Lancet. (2016) 387:2145–54. doi: 10.1016/S0140-6736(15)00516-4 26520231 PMC4848186

[2] Baird K Baillon S Lau LSL Storey M Lindesay J Velayudhan L. Predictive factors for conversion to dementia in individuals with early-onset mild cognitive impairment. Dement Geriatr Cogn Disord. (2021) 50:548–53. doi: 10.1159/000520882 34937020

[3] Campbell NL Unverzagt F LaMantia MA Khan BA Boustani MA. Risk factors for the progression of mild cognitive impairment to dementia. Clin Geriatr Med. (2013) 29:873–93. doi: 10.1016/j.cger.2013.07.009 24094301 PMC5915285

[4] Sperling RA Aisen PS Beckett LA Bennett DA Craft S Fagan AM . Toward defining the preclinical stages of Alzheimer's disease: recommendations from the National Institute on Aging-Alzheimer's Association workgroups on diagnostic guidelines for Alzheimer's disease. Alzheimers Dement. (2011) 7:280–92. doi: 10.1016/j.jalz.2011.03.003 21514248 PMC3220946

[5] Frisoni GB Altomare D Ribaldi F Villain N Brayne C Mukadam N . Dementia prevention in memory clinics: recommendations from the European task force for brain health services. Lancet Reg Health Eur. (2023) 26:100576. doi: 10.1016/j.lanepe.2022.100576 36895446 PMC9989648

[6] Livingston G Huntley J Sommerlad A Ames D Ballard C Banerjee S . Dementia prevention, intervention, and care: 2020 report of the Lancet Commission. Lancet. (2020) 396:413–46. doi: 10.1016/S0140-6736(20)30367-6 32738937 PMC7392084

[7] Norman K Haß U Pirlich M. Malnutrition in older adults-recent advances and remaining challenges. Nutrients. (2021) 13. doi: 10.3390/nu13082764 34444924 PMC8399049

[8] Dent E Wright ORL Woo J Hoogendijk EO. Malnutrition in older adults. Lancet. (2023) 401:951–66. doi: 10.1016/S0140-6736(22)02612-5 36716756

[9] Norton S Matthews FE Barnes DE Yaffe K Brayne C. Potential for primary prevention of Alzheimer's disease: an analysis of population-based data. Lancet Neurol. (2014) 13:788–94. doi: 10.1016/S1474-4422(14)70136-X 25030513

[10] Zhang X Yang L Wang Z Wang H Nie S Zhao C . Associations between nutritional status and cognitive impairment in older adults: results from the NHANES 2011–2014 cycles. Front Nutr. (2025) 12:1571990. doi: 10.3389/fnut.2025.1571990 40678779 PMC12267017

[11] Malara A Sgrò G Caruso C Ceravolo F Curinga G Renda GF . Relationship between cognitive impairment and nutritional assessment on functional status in Calabrian long-term-care. Clin Interv Aging. (2014) 9:105–10. doi: 10.2147/CIA.S54611 24453481 PMC3892960

[12] Volkert D Chourdakis M Faxen-Irving G Frühwald T Landi F Suominen MH . ESPEN guidelines on nutrition in dementia. Clin Nutr. (2015) 34:1052–73. doi: 10.1016/j.clnu.2015.09.004 26522922

[13] Ismail Z Black SE Camicioli R Chertkow H Herrmann N Laforce R . Recommendations of the 5th Canadian Consensus Conference on the diagnosis and treatment of dementia. Alzheimers Dement. (2020) 16:1182–95. doi: 10.1002/alz.12105 32725777 PMC7984031

[14] Bouillanne O Morineau G Dupont C Coulombel I Vincent J Nicolis I . Geriatric Nutritional Risk Index: a new index for evaluating at-risk elderly medical patients. Am J Clin Nutr. (2005) 82:777–83. doi: 10.1093/ajcn/82.4.777 16210706

[15] Takagi K Buettner S Ijzermans JNM. Prognostic significance of the controlling nutritional status (CONUT) score in patients with colorectal cancer: a systematic review and meta-analysis. Int J Surg. (2020) 78:91–6. doi: 10.1016/j.ijsu.2020.04.046 32335238

[16] Doi S Iwata H Wada H Funamizu T Shitara J Endo H . A novel and simply calculated nutritional index serves as a useful prognostic indicator in patients with coronary artery disease. Int J Cardiol. (2018) 262:92–8. doi: 10.1016/j.ijcard.2018.02.039 29706396

[17] Zhang Z Pereira SL Luo M Matheson EM. Evaluation of blood biomarkers associated with risk of malnutrition in older adults: a systematic review and meta-analysis. Nutrients. (2017) 9:829. doi: 10.3390/nu9080829 28771192 PMC5579622

[18] Nakamura MT Yudell BE Loor JJ. Regulation of energy metabolism by long-chain fatty acids. Prog Lipid Res. (2014) 53:124–44. doi: 10.1016/j.plipres.2013.12.001 24362249

[19] National Research Council Committee on Diet and Health. Diet and Health: Implications for Reducing Chronic Disease Risk. Washington (DC): National Academies Press (US) (1989). 25032333

[20] Gong J Harris K Peters SAE Woodward M. Serum lipid traits and the risk of dementia: a cohort study of 254,575 women and 214,891 men in the UK biobank. EClinicalMedicine. (2022) 54:101695. doi: 10.1016/j.eclinm.2022.101695 36247924 PMC9561731

[21] Eymundsdottir H Ramel A Geirsdottir OG Skuladottir SS Gudmundsson LS Jonsson PV . Body weight changes and longitudinal associations with cognitive decline among community-dwelling older adults. Alzheimers Dement. (2021) 13:e12163. doi: 10.1002/dad2.12163 PMC7896555 33665348

[22] Ceolin C Prinelli F Trevisan C Ravelli A Conti S Brennan L . Body weight trajectories from midlife are associated with cognitive decline in advanced age. Sci Rep. (2025) 15:24128. doi: 10.1038/s41598-025-08725-5 40619502 PMC12230133

[23] Buie JJ Watson LS Smith CJ Sims-Robinson C. Obesity-related cognitive impairment: the role of endothelial dysfunction. Neurobiol Dis. (2019) 132:104580. doi: 10.1016/j.nbd.2019.104580 31454547 PMC6834913

[24] Dimache AM alaru DL Sascau R Statescu C. The role of high triglycerides level in predicting cognitive impairment: a review of current evidence. Nutrients. (2021) 13:2118. doi: 10.3390/nu13062118 34203094 PMC8234148

[25] Huang R Pang Q Zheng L Lin J Li H Wan L . Cholesterol metabolism: physiological versus pathological aspects in intracerebral hemorrhage. Neural Regen Res. (2025) 20:1015–30. doi: 10.4103/NRR.NRR-D-23-01462 38989934 PMC11438341

[26] Zampelas A Magriplis E. New insights into cholesterol functions: a friend or an enemy? Nutrients. (2019) 11:1645. doi: 10.3390/nu11071645 31323871 PMC6682969

[27] Liu G Zhang J. Association of a novel nutritional index with cognitive impairment in middle-aged and elderly Chinese adults: a cross-sectional analysis from the China Health and Retirement Longitudinal Study. Front Nutr. (2025) 12:1486917. doi: 10.3389/fnut.2025.1486917 39963661 PMC11830621

[28] Fine JP Gray RJ A. Proportional hazards model for the subdistribution of a competing risk. J Am Stat Assoc. (1999) 94:496–509. doi: 10.1080/01621459.1999.10474144

[29] Zhao Y Hu Y Smith JP Strauss J Yang G. Cohort profile: the China Health and Retirement Longitudinal Study (CHARLS). Int J Epidemiol. (2014) 43:61–8. doi: 10.1093/ije/dys203 23243115 PMC3937970

[30] Wang Y Mo D Hu W Zhong Y Li Z Ma H . Comparison of novel nutritional index (TCBI) and insulin resistance index (TyG-BMI) for assessing cardiovascular disease risk: a cohort study. J Health Popul Nutr. (2026) 45:66. doi: 10.1186/s41043-025-01234-1 41578350 PMC12914969

[31] Levy R. Aging-associated cognitive decline. Working party of the International Psychogeriatric Association in collaboration with the World Health Organization. Int Psychogeriatr. (1994) 6:63–8. doi: 10.1017/S1041610294001626 8054494

[32] Bai A Shi H Huang X Xu W Deng Y. Association of C-reactive protein and motoric cognitive risk syndrome in community-dwelling older adults: the China Health and Retirement Longitudinal Study. J Nutr Health Aging. (2021) 25:1090–5. doi: 10.1007/s12603-021-1678-3 34725666 PMC12929984

[33] Jak AJ Bondi MW Delano-Wood L Wierenga C Corey-Bloom J Salmon DP . Quantification of five neuropsychological approaches to defining mild cognitive impairment. Am J Geriatr Psychiatry. (2009) 17:368–75. doi: 10.1097/JGP.0b013e31819431d5 19390294 PMC2743175

[34] Richards M Touchon J Ledesert B Richie K. Cognitive decline in ageing: are AAMI and AACD distinct entities? Int J Geriatr Psychiatry. (1999) 14:534–40. doi: 10.1002/(SICI)1099-1166(199907)14:7<534::AID-GPS963>3.0.CO;2-B 10440973

[35] Cui Y Xu Z Cui Z Guo Y Wu P Zhou X. Comparative study of insulin resistance surrogate indices to predict mild cognitive impairment among Chinese non-diabetic adults. Lipids Health Dis. (2024) 23:357. doi: 10.1186/s12944-024-02353-0 39487494 PMC11529243

[36] D'Amico G Morabito A D'Amico M Pasta L Malizia G Rebora P . Clinical states of cirrhosis and competing risks. J Hepatol. (2018) 68:563–76. doi: 10.1016/j.jhep.2017.10.020 29111320

[37] Austin PC Fine JP. Practical recommendations for reporting Fine-Gray model analyses for competing risk data. Stat Med. (2017) 36:4391–400. doi: 10.1002/sim.7501 28913837 PMC5698744

[38] Jiang L Zhu T Song W Zhai Y Tang Y Ruan F . Assessment of six insulin resistance surrogate indexes for predicting stroke incidence in Chinese middle-aged and elderly populations with abnormal glucose metabolism: a nationwide prospective cohort study. Cardiovasc Diabetol. (2025) 24:56. doi: 10.1186/s12933-025-02618-7 39915878 PMC11804005

[39] Yue Y Li P Sun Z Murayama R Li Z Hashimoto K . Association of novel triglyceride-glucose-related indices with incident stroke in early-stage cardiovascular-kidney-metabolic syndrome. Cardiovasc Diabetol. (2025) 24:301. doi: 10.1186/s12933-025-02854-x 40707904 PMC12291248

[40] Borges MK Cezar NOdC Siqueira ASS Yassuda M Cesari M Aprahamian I. The relationship between physical frailty and mild cognitive impairment in the elderly: a systematic review. J Frailty Aging. (2019) 8:192–7. doi: 10.14283/jfa.2019.29 31637405 PMC12275732

[41] Altman DG Bland JM. Interaction revisited: the difference between two estimates. BMJ. (2003) 326:219. doi: 10.1136/bmj.326.7382.219 12543843 PMC1125071

[42] Xiao B Zhu J Han Y. Association of a novel nutritional marker, the triglyceride-cholesterol-body weight index, with 90-day unfavorable outcomes in acute ischemic stroke: a prospective cohort study. Front Nutr. (2025) 12:1707231. doi: 10.3389/fnut.2025.1707231 41567332 PMC12815849

[43] Li G Li S. Exploring the prognostic value of the novel nutritional index for in-hospital mortality in acute coronary syndrome: a sex-specific analysis. Front Med. (2025) 12:1498260. doi: 10.3389/fmed.2025.1498260 PMC12058770 40342584

[44] Sun HL . A novel nutritional index based on triglyceride, cholesterol, and body weight as a protective factor against sarcopenia: evidence from CHARLS and NHANES. Nutr Hosp. (2026). doi: 10.20960/nh.06151 41878922

[45] Otsuka R. Diet, nutrition, and cognitive function: a narrative review of Japanese longitudinal studies. Geriatr Gerontol Int. (2022) 22:825–31. doi: 10.1111/ggi.14463 36002912 PMC9805113

[46] Bourre JM. The role of nutritional factors on the structure and function of the brain: an update on dietary requirements. Rev Neurol. (2004) 160:767–92. doi: 10.1016/s0035-3787(04)71032-2 15454864

[47] Lu J Guo QQ Wang Y Li YY Zuo ZX. The evolutionary stage of cognitive frailty and its changing characteristics in old adults. J Nutr Health Aging. (2021) 25:467–78. doi: 10.1007/s12603-020-1560-8 33786564 PMC12876665

[48] Chye L Wei K Nyunt MS Gao Q Wee SL Ng T. Strong relationship between malnutrition and cognitive frailty in the Singapore longitudinal ageing studies (SLAS-1 and SLAS-2). J Prev Alzheimers Dis. (2018) 5:142–8. doi: 10.14283/jpad.2017.46 29616708 PMC12280758

[49] Cereda E Pedrolli C Klersy C Bonardi C Quarleri L Cappello S . Nutritional status in older persons according to healthcare setting: a systematic review and meta-analysis of prevalence data using MNA^®^. Clin Nutr. (2016) 35:1282–90. doi: 10.1016/j.clnu.2016.03.008 27086194

[50] Başar GB Canpolat EB Budán F Agagündüz D Szép D. Nutritional and physical pathways from feeding difficulty to sarcopenia in Alzheimer's dementia. Front Nutr. (2025) 12:1740327. doi: 10.3389/fnut.2025.1740327 41601867 PMC12832365

[51] Güner M Baş AO Ceylan S Kahyaoglu Z Çöteli S Ünsal P . Dysphagia is closely related to frailty in mild-to-moderate Alzheimer's disease. BMC Geriatr. (2023) 23:304. doi: 10.1186/s12877-023-04020-y 37198547 PMC10189997

[52] Fekete M Varga P Ungvari Z Fekete JT Buda A Szappanos Á . The role of the Mediterranean diet in reducing the risk of cognitive impairement, dementia, and Alzheimer's disease: a meta-analysis. Geroscience. (2025) 47:3111–30. doi: 10.1007/s11357-024-01488-3 39797935 PMC12181514

[53] Cai D Zeng Y Xu X Ye M Song A Chen M. Association of the cMIND diet with cognitive impairment in older adults: evidence from a 10-year nationwide study. Front Nutr. (2025) 12:1716435. doi: 10.3389/fnut.2025.1716435 41608510 PMC12834752

[54] Lu Y Pan F Gwee X Chua DQ Ng TP Lee TS . Nutritional status and risks of cognitive decline and incident neurocognitive disorders: Singapore longitudinal ageing studies. J Nutr Health Aging. (2021) 25:660–7. doi: 10.1007/s12603-021-1603-9 33949634 PMC12280686

[55] Wang X Xin Z Li X Wu K Wang W Guo L . Mediterranean diet and dementia: MRI marker evidence from meta-analysis. Eur J Med Res. (2025) 30:32. doi: 10.1186/s40001-025-02276-1 39815306 PMC11737277

[56] Reuben DB Kremen S Maust DT. Dementia prevention and treatment: a narrative review. JAMA Intern Med. (2024) 184:563–72. doi: 10.1001/jamainternmed.2023.8522 38436963

[57] Schüler R Seebeck N Osterhoff MA Witte V Flöel A Busjahn A . VEGF and GLUT1 are highly heritable, inversely correlated and affected by dietary fat intake: consequences for cognitive function in humans. Mol Metab. (2018) 11:129–36. doi: 10.1016/j.molmet.2018.02.004 29506909 PMC6001408

[58] Zhang Q Jin K Chen B Liu R Cheng S Zhang Y . Overnutrition induced cognitive impairment: insulin resistance, gut-brain axis, and neuroinflammation. Front Neurosci. (2022) 16:884579. doi: 10.3389/fnins.2022.884579 35873818 PMC9298971

[59] Victor MB Leary N Luna X Meharena HS Scannail AN Bozzelli PL . Lipid accumulation induced by APOE4 impairs microglial surveillance of neuronal-network activity. Cell Stem Cell. (2022) 29:1197–212.e8. doi: 10.1016/j.stem.2022.07.005 35931030 PMC9623845

[60] Liu H Zou L Zhou R Zhang M Gu S Zheng J . Long-term increase in cholesterol is associated with better cognitive function: evidence from a longitudinal study. Front Aging Neurosci. (2021) 13:691423. doi: 10.3389/fnagi.2021.691423 34220488 PMC8248815

[61] Power MC Rawlings A Sharrett AR Bandeen-Roche K Coresh J Ballantyne CM . Association of midlife lipids with 20-year cognitive change: a cohort study. Alzheimers Dement. (2018) 14:167–77. doi: 10.1016/j.jalz.2017.07.757 28916238 PMC5803364

[62] Iwagami M Qizilbash N Gregson J Douglas I Johnson M Pearce N . Blood cholesterol and risk of dementia in more than 1·8 million people over two decades: a retrospective cohort study. Lancet Healthy Longev. (2021) 2:e498–506. doi: 10.1016/S2666-7568(21)00150-1 36097999

[63] Aine CJ Sanfratello L Adair JC Knoefel JE Qualls C Lundy SL . Characterization of a normal control group: are they healthy? Neuroimage. (2014) 84:796–809. doi: 10.1016/j.neuroimage.2013.09.025 24060318 PMC3892768

[64] Dunk MM Driscoll I. Total Cholesterol and APOE-related risk for Alzheimer's disease in the Alzheimer's disease neuroimaging initiative. J Alzheimers Dis. (2022) 85:1519–28. doi: 10.3233/JAD-215091 34958023 PMC10442640

[65] Jarvik GP Wijsman EM Kukull WA Schellenberg GD Yu C Larson EB. Interactions of apolipoprotein E genotype, total cholesterol level, age, and sex in prediction of Alzheimer's disease: a case-control study. Neurology. (1995) 45:1092–6. doi: 10.1212/WNL.45.6.1092 7783869

[66] Heverin M Meaney S Lütjohann D Diczfalusy U Wahren J Björkhem I. Crossing the barrier: net flux of 27-hydroxycholesterol into the human brain. J Lipid Res. (2005) 46:1047–52. doi: 10.1194/jlr.M500024-JLR200 15741649

[67] Lee LL Aung HH Rutledge JC Rutkowsky JM Wilson DW Anderson SE. Triglyceride-rich lipoprotein lipolysis products increase blood-brain barrier transfer coefficient and induce astrocyte lipid droplets and cell stress. Am J Physiol Cell Physiol. (2017) 312:C500–16. doi: 10.1152/ajpcell.00120.2016 28077357 PMC5407020

[68] McFadden T Musaus M Nelsen JL Martin K Jones N Smith P . Dysregulation of protein degradation in the hippocampus is associated with impaired spatial memory during the development of obesity. Behav Brain Res. (2020) 393:112787. doi: 10.1016/j.bbr.2020.112787 32603798 PMC7423702

[69] Farr SA Yamada KA Butterfield DA Abdul HM Xu L Miller NE . Obesity and hypertriglyceridemia produce cognitive impairment. Endocrinology. (2008) 149:2628–36. doi: 10.1210/en.2007-1722 18276751 PMC2329289

[70] Zhou Z Ryan J Tonkin AM Zoungas S Lacaze P Wolfe R . Association between triglycerides and risk of dementia in community-dwelling older adults: a prospective cohort study. Neurology. (2023) 101:e2288–99. doi: 10.1212/WNL.0000000000207923 37879942 PMC10727221

[71] Parthasarathy V Frazier DT Bettcher BM Jastrzab L Chao L Reed B . Triglycerides are negatively correlated with cognitive function in nondemented aging adults. Neuropsychology. (2017) 31:682–8. doi: 10.1037/neu0000335 28604016 PMC5726405

[72] Sims RC Madhere S Gordon S Clark E Jr Abayomi KA Callender CO . Relationships among blood pressure, triglycerides and verbal learning in African Americans. J Natl Med Assoc. (2008) 100:1193–8. doi: 10.1016/S0027-9684(15)31474-7 18942281 PMC3804020

[73] Raffaitin C Gin H Empana JP Helmer C Berr C Tzourio C . Metabolic syndrome and risk for incident Alzheimer's disease or vascular dementia: the three-city study. Diabetes Care. (2009) 32:169–74. doi: 10.2337/dc08-0272 18945929 PMC2606808

[74] Reitz C Luchsinger J Tang M- Manly J Mayeux R. Impact of plasma lipids and time on memory performance in healthy elderly without dementia. Neurology. (2005) 64:1378–83. doi: 10.1212/01.WNL.0000158274.31318.3C 15851727 PMC2737324

[75] Zhu Y Liu X Zhu R Zhao J Wang Q. Lipid levels and the risk of dementia: a dose-response meta-analysis of prospective cohort studies. Ann Clin Transl Neurol. (2022) 9:296–311. doi: 10.1002/acn3.51516 35202496 PMC8935316

