# Supplementary material for: Association of a novel nutritional index, the triglyceride-cholesterol-body weight index (TCBI), with incident mild cognitive impairment: CHARLS 2011–2018
Source: Front Nutr. 2026 Jun 4;13:1834512. doi: 10.3389/fnut.2026.1834512 (PMC13275355; doi:10.3389/fnut.2026.1834512)
Supplement: Supplementary file 1 [file Table_1.docx]

**Supplementary Table 1.Age-stratified cutoff values for MCI based on composite cognitive scores according to the AACD criteria**

| **Age**  **(Years)** | **Sample Size**  **(n)** | **Cognition Score**  **(Mean ± SD)** | **Threshold** |
| --- | --- | --- | --- |
| **<60** | 6922 | 16.79 ± 4.56 | 12.23 |
| **60-64** | 1986 | 15.80 ± 4.51 | 11.29 |
| **65-69** | 1285 | 15.21 ± 4.73 | 10.48 |
| **70-74** | 803 | 14.06 ± 4.82 | 9.24 |
| **75-79** | 446 | 12.73 ± 4.74 | 7.99 |
| **80+** | 200 | 11.25 ± 5.14 | 6.11 |
| **Total** | 11642 | 16.01 ± 4.77 | 11.24 |

Note: Composite cognitive score refers to the baseline global cognitive score of the available study sample, calculated within each age stratum and presented as mean ± standard deviation (SD). Thresholds represent the age-specific cutoff values for mild cognitive impairment (MCI) determined according to the Age-Associated Cognitive Decline (AACD) criteria and were used to define the MCI outcome in the present study. To reduce potential selection bias, these thresholds were calculated and fixed using the full available sample before applying other necessary exclusion criteria, and were then kept unchanged in all subsequent analyses. Sample size indicates the number of participants in each age group who were available for threshold calculation.

**Supplementary Table 2. Multicollinearity diagnostics for covariates in Model 3: variance inflation factors (VIFs).**

| Term | VIF |
| --- | --- |
| TCBl_ | 1.498887 |
| Sex | 2.505899 |
| Age | 1.500726 |
| Marital status | 1.122793 |
| Education level | 1.172256 |
| Place of residence | 1.056610 |
| Smoking status | 2.142721 |
| Alcohol consumption status | 1.529901 |
| Social participation | 1.020569 |
| Nighttime sleep duration | 1.070888 |
| Hypertension | 2.007349 |
| Stroke | 1.021026 |
| Cardiac disease | 1.045230 |
| DBP | 2.625676 |
| FPG.mg.dL | 1.630690 |
| HDL-C.mg.dL | 1.308329 |
| LDL-C.mg.dL | 1.199700 |
| Diabetes | 1.617568 |
| Depressive.symptoms | 1.107113 |
| SBP | 3.399498 |

**Supplementary Table 3.Missing data distribution for study variables (counts and percentages based on the total sample size of 5,510)**

| **Variable** | **Missing Count** | **Missing Percentage** |
| --- | --- | --- |
| **Alcohol consumption status** | 1 | 0.018% |
| **Hypertension** | 28 | 0.508% |
| **Diabetes** | 76 | 1.379% |
| **Dyslipidemia** | 46 | 0.834% |
| **BMI** | 12 | 0.217% |
| **DBP,mmHg** | 19 | 0.344% |
| **SBP,mmHg** | 35 | 0.635% |
| **HDL-C,mg/dL** | 2 | 0.036% |
| **LDL-C,mg/dL** | 7 | 0.127% |
| **FPG,mg/dL** | 5 | 0.091% |
| **HbA1C,%** | 38 | 0.689% |
| **Nighttime sleep duration** | 18 | 0.326% |
| **Smoking status** | 12 | 0.218% |
| **Cardiac disease** | 31 | 0.563% |
| **Stroke** | 42 | 0.762% |
| **Depressive symptoms** | 19 | 0.344% |
| **WC** | 43 | 0.780% |

**Supplementary Table 4. Association between baseline TCBI and loss to follow-up: Cox proportional hazards models for informative censoring assessment**

| **Characteristic** | **Model 1** | | | **Model 2** | | | **Model 3** | | |
| --- | --- | --- | --- | --- | --- | --- | --- | --- | --- |
|  | **HR** | **95% CI** | **p-value** | **HR** | **95% CI** | **p-value** | **HR** | **95% CI** | **p-value** |
| **TCBI (continuous)** | 0.88 | 0.73, 1.07 | 0.195 | 0.92 | 0.76, 1.12 | 0.409 | 0.84 | 0.66, 1.06 | 0.140 |
| **TCBI** |  |  |  |  |  |  |  |  |  |
| Q1 | — | — |  | — | — |  | — | — |  |
| Q2 | 0.88 | 0.75, 1.04 | 0.142 | 0.90 | 0.76, 1.05 | 0.184 | 0.88 | 0.74, 1.04 | 0.132 |
| Q3 | 0.91 | 0.78, 1.07 | 0.268 | 0.92 | 0.78, 1.08 | 0.311 | 0.88 | 0.73, 1.05 | 0.159 |
| Q4 | 0.92 | 0.79, 1.09 | 0.339 | 0.95 | 0.81, 1.12 | 0.549 | 0.90 | 0.74, 1.09 | 0.269 |
| P for trend |  |  | 0.423 |  |  | 0.629 |  |  | 0.313 |
| CI, confidence interval; HR, hazard ratio; loss to follow-up was defined as participants who did not develop MCI or die during follow-up and who discontinued follow-up or had no further follow-up records before the planned end of follow-up (i.e., censored participants for whom the reason for censoring was unrelated to an observed outcome). TCBI, triglyceride-cholesterol-body weight index. TCBI was log10-transformed before analysis.  Model 1: unadjusted. Model 2: adjusted for age and sex. Model 3: additionally adjusted for marital status, education level, place of residence, smoking status, alcohol consumption status, social participation, nighttime sleep duration, hypertension, diabetes, stroke, cardiac disease, depressive symptoms, systolic blood pressure (SBP), diastolic blood pressure (DBP), fasting plasma glucose, HDL-C, and LDL-C. | | | | | | | | | |

**Baseline characteristics of participants lost to follow-up versus the remaining participants (assessment of differential loss to follow-up)**

| **Characteristic** |  | |  | |
| --- | --- | --- | --- | --- |
|  | **Remaining participants  N = 4,363** | **Loss to follow-up N = 1,147** | **SMD** | **95% CI** |
| **Age (years)** | 58 ± 9 | 60 ± 9 | -0.23 | -0.29, -0.16 |
| **Sex** |  |  | 0.07 | 0.01, 0.14 |
| Male | 2,429 (55.7%) | 597 (52.0%) |  |  |
| Female | 1,934 (44.3%) | 550 (48.0%) |  |  |
| **Place of residence** | 2,630 (60.3%) | 636 (55.4%) | 0.10 | 0.03, 0.16 |
| **Marital status** |  |  | 0.10 | 0.03, 0.16 |
| Married | 3,957 (90.7%) | 1,006 (87.7%) |  |  |
| Other | 406 (9.3%) | 141 (12.3%) |  |  |
| **Education level** |  |  | 0.33 | 0.26, 0.39 |
| No formal education | 1,220 (28.0%) | 497 (43.3%) |  |  |
| Primary/middle school | 2,469 (56.6%) | 518 (45.2%) |  |  |
| High school or above | 674 (15.4%) | 132 (11.5%) |  |  |
| **Smoking status** |  |  | 0.06 | 0.00, 0.13 |
| Current smoker | 1,502 (34.4%) | 368 (32.1%) |  |  |
| Former smoker | 467 (10.7%) | 113 (9.9%) |  |  |
| Never smoker | 2,394 (54.9%) | 666 (58.1%) |  |  |
| **Alcohol consumption status** |  |  | 0.06 | 0.00, 0.13 |
| Current drinker | 1,633 (37.4%) | 395 (34.4%) |  |  |
| Former drinker | 375 (8.6%) | 104 (9.1%) |  |  |
| Never drinker | 2,355 (54.0%) | 648 (56.5%) |  |  |
| **Social participation** |  |  | 0.00 | -0.06, 0.07 |
| Yes | 2,468 (56.6%) | 650 (56.7%) |  |  |
| No | 1,895 (43.4%) | 497 (43.3%) |  |  |
| **Stroke** |  |  | 0.02 | -0.05, 0.08 |
| Yes | 81 (1.9%) | 19 (1.7%) |  |  |
| No | 4,282 (98.1%) | 1,128 (98.3%) |  |  |
| **Cardiac disease** |  |  | 0.01 | -0.06, 0.07 |
| Yes | 524 (12.0%) | 141 (12.3%) |  |  |
| No | 3,839 (88.0%) | 1,006 (87.7%) |  |  |
| **Hypertension** |  |  | 0.08 | 0.02, 0.15 |
| Yes | 1,736 (39.8%) | 504 (43.9%) |  |  |
| No | 2,627 (60.2%) | 643 (56.1%) |  |  |
| **Dyslipidemia** |  |  | 0.01 | -0.05, 0.08 |
| Yes | 2,194 (50.3%) | 569 (49.6%) |  |  |
| No | 2,169 (49.7%) | 578 (50.4%) |  |  |
| **Diabetes** |  |  | 0.05 | -0.02, 0.11 |
| Yes | 734 (16.8%) | 213 (18.6%) |  |  |
| No | 3,629 (83.2%) | 934 (81.4%) |  |  |
| **Depressive symptoms** |  |  | 0.01 | -0.06, 0.07 |
| Yes | 1,352 (31.0%) | 359 (31.3%) |  |  |
| No | 3,011 (69.0%) | 788 (68.7%) |  |  |
| **Antihypertensive agents** |  |  | 0.05 | -0.01, 0.12 |
| Yes | 873 (20.0%) | 253 (22.1%) |  |  |
| No | 3,490 (80.0%) | 894 (77.9%) |  |  |
| **Antihyperlipidemic agents** |  |  | ＜0.01 | -0.06, 0.07 |
| Yes | 262 (6.0%) | 69 (6.0%) |  |  |
| No | 4,101 (94.0%) | 1,078 (94.0%) |  |  |
| **SBP** | 130 ± 21 | 132 ± 21 | -0.12 | -0.18, -0.05 |
| **DBP** | 76 ± 12 | 76 ± 12 | -0.03 | -0.09, 0.04 |
| **BMI** | 23.9 ± 3.9 | 23.8 ± 4.0 | 0.03 | -0.04, 0.09 |
| **Body weight,Kg** | 61 ± 12 | 60 ± 11 | 0.12 | 0.06, 0.19 |
| **Body height,cm** | 160 ± 8 | 159 ± 8 | 0.16 | 0.09, 0.22 |
| **WC,cm** | 85 ± 13 | 85 ± 12 | -0.01 | -0.07, 0.06 |
| **TG,mg/dL** | 107 (76, 158) | 108 (75, 160) | 0.01 | -0.06, 0.07 |
| **TC,mg/dL** | 194 ± 38 | 194 ± 39 | ＜0.01 | -0.06, 0.07 |
| **FPG,mg/dL** | 103 (95, 114) | 102 (94, 114) | -0.02 | -0.09, 0.04 |
| **HbA1C,%** | 5.10 (4.90, 5.40) | 5.10 (4.90, 5.40) | 0.01 | -0.06, 0.07 |
| **HDL-C,mg/dL** | 50 ± 15 | 50 ± 15 | ＜0.01 | -0.06, 0.07 |
| **LDL-C,mg/dL** | 117 ± 35 | 117 ± 36 | ＜0.01 | -0.06, 0.07 |
| **Nighttime sleep duration** | 6.46 ± 1.70 | 6.38 ± 1.78 | 0.04 | -0.02, 0.11 |
| **Lg TCBI** | 3.11 ± 0.31 | 3.10 ± 0.31 | 0.04 | -0.03, 0.10 |

SMD, standardized mean difference; CI, confidence interval; SBP, systolic blood pressure; DBP, diastolic blood pressure; BMI, body mass index; WC, waist circumference; TG, triglycerides; TC, total cholesterol; FPG, fasting plasma glucose; HbA1c, glycated hemoglobin; HDL-C, high-density lipoprotein cholesterol; LDL-C, low-density lipoprotein cholesterol; Lg TCBI, log10-transformed triglyceride-cholesterol-body weight index.
